# Animal Poisons as Causes of Diseases; Indications for Treatment in Diseases Produced by Them

**Published:** 1864-03

**Authors:** N. S. Davis

**Affiliations:** Professor of Practical and Clinical Medicine


ARTICLE XI.

### ANIMAL POISONS AS CAUSES OF DISEASES; INDICATIONS FOR TREATMENT IN DISEASES PRODUCED BY THEM.

BY N. S. DAVIS, M.D., Prof. of Practical and Clinical Medicine.

Remarks made in a Discussion before the Chicago Medical Society, Feb. 19th, 1864.

The question before the Society for discussion this evening, is, what are the indications for treatment in diseases arising from animal poisons? The time allotted to each member, under the rules of the Society, is so limited as to preclude all preliminary remarks in relation to the existence and *modus operandi* of animal poisons, and compels me to proceed directly to the question. Assuming, then, that animal poisons exist capable of producing disease; and that they act through the medium of the blood, to which they gain access, by absorption from some injured or diseased surface; by inhalation with the air through the lungs; or by retention, through failure of some one of the excretory organs to perform its functions, I will proceed directly to the question. The distinct rational indications for treatment in diseases of the class here supposed, may be stated in four propositions:—

*First*, To neutralize or antidote the poison, and thereby render it harmless.

*Second*, To introduce such remedial agents as will retard or altogether stop the SEPTIC or deteriorating influence that most of the animal poisons produce on the constituents of the blood.

*Third*, To favor, as far as possible, the elimination of the poison through the natural excretory organs.

*Fourth*, To mitigate or remove the irritating effects of the poison, on the properties and functions of the organized tissues of the body.

If the first of these indications could be fulfilled completely, during the incipient stage of the disease, it would doubtless remedy the whole difficulty, and render the remaining indications unnecessary.

When the poison is being introduced by absorption from wounds or injured parts, as the bites of serpents, rabid animals, &c.; or from erysipelatous, gangrenous, and suppurating surfaces, such a result is sometimes practicable, by the application of the antidote directly to the wounds or diseased surfaces.

The practicability of this has been many times demonstrated by the speedy application of a solution of iodine to the bites of poisonous serpents, and more recently by the local application of bromine, &c., to tissues in a state of gangrene. A variety of facts seem to show conclusively that iodine, chlorine, and bromine are reliable antidotes to many, at least, of the animal poisons, wherever they can be brought fully in contact with them.

But each of them possesses properties of so much activity, when taken internally, that they cannot be safely introduced into the blood in sufficient quantity to act as efficient antidotes to animal poisons already impregnating the mass of that fluid.

Conceding the fact, then, that we are yet unacquainted with any therapeutic agents what can be safely trusted to antidote animal poisons already in the blood, the second indication named becomes of much practical importance; for if we can introduce into the blood, through absorption from the stomach, any agent or agents, the presence of which will prevent the poison from inducing its special *septic*, or peculiar change, in the quality of the blood and the properties of the tissues, it will sooner or later find an exit from the system through some of the excretory organs, and the convalescence of the patient will have been secured. It is unquestionably true that most of the animal poisons produce their violent and dangerous effects, by primarily acting on the constituents of the blood and properties of tissues in such a way as to effect a more or less rapid increase of themselves. Thus the most minute atom of variolous poison, introduced into the system, either by inoculations or inhalation, produces no perceptible disturbance for several days, when it is found to have so multiplied as to furnish deposits in the whole cutaneous surface, and even to impregnate the surrounding air to a certain extent. So, too, in the absorption of pus or putrid animal matter from an unhealthy suppurating surface, or from phlebitic inflammation, the minute quantity thus absorbed becomes rapidly multiplied until a general state of pyœmia is induced and puruloid deposits are found in various parts of the system, with a rapid exhaustion of the patient.

It is this change in the elements of the blood and the properties of the tissues that constitutes the *septic* or zymotic action of animal poisons. To prevent or arrest this action, so as to avoid the multiplication of the poison in the system, constitutes the second indication for treatment, as already mentioned. The remedies that have long been used for fulfilling this indication, though without a very definite idea of their *modus operandi*, are the mineral acids; the preparations of Peruvian bark; and the chlorine salts, more especially, the acid tincture of chloride of iron. More recently the experiments of Dr. Polli, in Europe, showing the efficiency of the sulphites of lime and soda, in preventing the injurious effects of pus and putrid animal matter when introduced into the blood of animals, has caused those substances to be used as remedies in a variety of diseases supposed to depend on blood poisoning. From my own experience thus far, I am led to believe they will be found to possess great value under certain circumstances. But it will be chiefly as *anti-septics*, in preventing the action of such animal poisons as are derived from the decomposition of animal matter; and to be effectual they must be administered sufficiently early to be present in the blood coincidently with, or very soon after, the introduction of the poison. It will be remembered, that Dr. POLLI, in some of his most striking experiments, administered the sulphites to the animals long enough to have them absorbed, before the poisonous matter was injected into the veins. And we are inclined to think that these substances will be found of very great value, whenever they can be administered sufficiently early to be present in the blood, before the poison has commenced its deteriorating effects on that fluid. For this purpose their administration should be commenced, whenever possible, during the incubative stage of such diseases as arise from the action of animal poisons. The quantity given should be sufficient to impregnate the fluids of the body freely, and the administration should be continued until all direct action of the poison ceases. It is highly probable that future experiments and observations will show that the action of the sulphites is most efficient against that class of animal poisons which arise chiefly from the decomposition of animal matter, and gives rise to erysipelas, phlebitis, and pyemia. Some have objected to the use of the sulphites, and all other remedies founded on the indication now under discussion, on the ground that the indication itself involves a humoral pathology, analagous to the ancient doctrines of concoction and fermentation; processes which they claim cannot take place in the living system. We see no force in the objection. For if we admit the absorption of an animal poison, it makes no practical difference in the indication for treatment, whether the poison produces its deteriorating effect by acting *directly* on the composition and properties of the blood, or *indirectly*, by first changing the vital properties of the solids, and through them affecting the quality of the fluids. Some of the remedies that have long been used in the class of diseases under consideration, such as the tincture of the choride of iron, undoubtedly act more or less on the vital affinity of the organized structures, and not merely by diminishing the alkalinity of the blood as many suppose.

The third indication we have named, is founded on the fact, that nearly all poisons manifest an affinity for some one or more of the exeretory structures or tissues through which they are ultimately eliminated, if life is preserved, and no antidotes are successfully administered. Thus, the contagions giving rise to the class of eruptive fevers, all manifest a strong affinity for the cutaneous tissue, in which they find a lodgement, induce specific inflammation, and from which they are finally eliminated. Whenever such a tendency can be discovered, it is obviously desirable, not only to carefully avoid such remedies as would interfere with the natural tendency of the poison, by producing active determinations in an opposite direction, but to use such as will gently promote the action of the organs most likely to eliminate the morbific agent. This rule would forbid the use of active cathartics and depletives in the early stage of all eruptive fevers, and clinical experience has fully demonstrated their inutility.

The fourth and last indication, calls for the use of such means as are calculated to allay the general excitement, and mitigate the extent and severity of local complications. If either one of the three preceding indications could be early and perfectly fulfilled, this last would be rendered unnecessary in most instances. But in the present state of our knowledge, this can seldom be done; and hence to mitigate the effects of a poison that he can neither completely antidote nor expel from the system, often becomes the paramount duty of the practitioner at the bedside. Of course, the remedies for this purpose, will be as variable as the effects to be treated. In some cases the active grade of excitement will call for sedatives and alterants; while in others stimulants, anodynes, and tonics may be required. Of course no individual remedies can be enumerated under this head, without entering into the details of each individual case that might be presented to the practitioner.

